# Supplementary material for: Photo-excited extracellular electron transfer of electroactive microorganism triggers RAFT polymerization
Source: Nat Commun. 2025 Nov 21;16:10257. doi: 10.1038/s41467-025-65119-x (PMC12638906; doi:10.1038/s41467-025-65119-x)
Supplement: Supplementary file 1 — Supplementary Information [file 41467_2025_65119_MOESM1_ESM.pdf]

Supplementary information (SI)

Photo-excited extracellular electron transfer of electroactive microorganism triggers RAFT polymerization

Chao Li[1, 2, 3, †], Jing Liu[4, †], Wenchang Hu[2, 3], Lin Xiao[2, 3], Feng Li[2, 3], Qijing Liu[2, 3], Junqi Zhang[1], Huan Yu[1], Baocai Zhang[1], Dake Xu[5, 6], Shaoan Cheng[7], Wen-Wei Li[8], Kenneth H. Nealson[9], and Hao Song[1, 2, 3, *]

[1] College of Life and Health Sciences, Northeastern University, Shenyang, 110169, China

[2] State Key Laboratory of Synthetic Biology, Tianjin University, Tianjin, 300072, China

[3] School of Synthetic Biology and Biomanufacturing, Tianjin University, Tianjin, 300072, China

[4] Institute of Entomology, College of Life Sciences, Nankai University, Tianjin, 300071, China

[5] Shenyang National Laboratory for Materials Science, Northeastern University, Shenyang, 110819, China

[6] Electrobiomaterials Institute, Key Laboratory for Anisotropy and Texture of Materials (Ministry of Education), Northeastern University, Shenyang, 110819, China

[7] State Key Laboratory of Clean Energy, Department of Energy Engineering, Zhejiang University, Hangzhou, 310027, China

[8] Chinese Academy of Sciences Key Laboratory of Urban Pollutant Conversion, Department of Environmental Science and Engineering, University of Science & Technology of China, Hefei, 230026, China

[9] Departments of Earth Science & Biological Sciences, University of Southern California, 4953 Harriman Ave., South Pasadena, CA 91030, USA

[†] These authors contributed equally: Chao Li, Jing Liu.

[*] To whom correspondence should be addressed. E-mail: songhao@mail.neu.edu.cn

Materials

The oxidation state riboflavin (R434261, RF, 98%) and riboflavin 5'-monophosphate sodium hydrate (F610334, FMN, 97%) were purchased from Aladdin Reagents. N, N-dimethylacrylamide (N159036, DMA, 98%), Poly (ethylene glycol) methyl ether acrylate (P133111, PEGA, average Mn = 480 g/mol), 2-Hydroxyethyl methacrylate (H103044, HEMA, 99%), Methacrylatoethyl trimethyl ammonium chloride (M102201, TMAEMA, 75 wt. % in H_2O), 2-Aminoethyl methacrylate hydrochloride (A168938, AOMA, 90%), 4-Acryloylmorpholine (A151729, AML, 98%), Sodium methacrylate (S103167, MAA, 99%), and Methyl methacrylate (M109623, MMA, 99%) were purchased from Aladdin Reagents. N-isopropylacrylamide (M86179, NIPAM, 98%) was purchased from Meryer Reagents. Liquid monomers were filtered over a column of basic alumina (Al_2O_3) to remove the polymerization inhibitor and stored at 4°C prior to use. Thiocarbonylthio compounds: 2-ethylsulfanylthiocarbonylsulfanyl-propionic acid methyl ester (AK0507, CTA1, AK Biotech, 98%), 4-Cyano-4-[(ethylsulfanylthiocarbonyl)sulfanyl] pentanoic acid (A14265, CTA2, Innochem, 97%), *S,S*'-bis(α,α'- dimethyl-α''-acetic acid) trithiocarbonate (T4240, CTA3, Innochem, 97%), Ethyl 2-ethoxycarbothioylsulfanylacetate (T14517, CTA4, Innochem, 97%), and Sodium hydrosulfite (A81152, $Na_2O_4S_2$, Innochem, 85%) were all used as received. Ultrapure water was produced using a Purelab flex milli-Q water purification system and sodium nitrate (S111648, Innochem, 99%) was used as received for GPC analysis. Deuterium oxide (D113904, D_2O, Aladdin, 99.9%) and Deutero N-Nitrosodimethylamine (N396014, Aladdin, DMSO-d6) were used as received for NMR analysis.

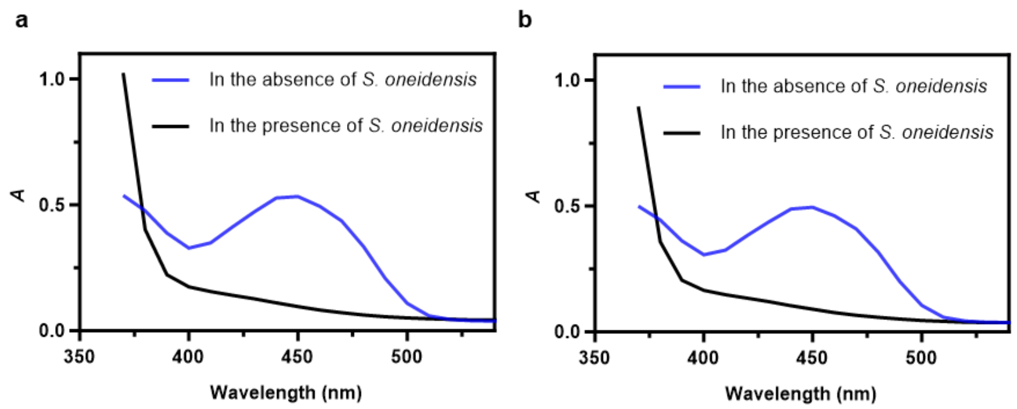

Supplementary Fig. 1 UV-vis absorption spectra of riboflavin (**a**) and FMN (**b**) in the absence and presence of *S. oneidensis* (the solutions were degassed with N_2). [flavin] = 8 μM. Source data were provided as a Source Data file.

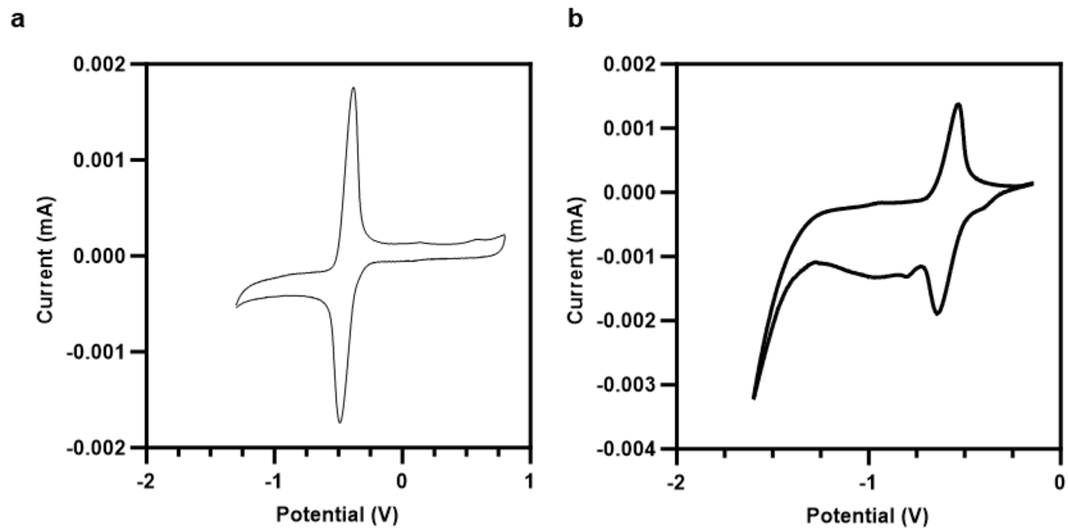

Supplementary Fig. 2 Cyclic voltammogram of RF to determine redox potential. Note: (**a**) the analysis was performed using PBK (0.1 M) as electrolyte in water ([RF] = 10^{-3} M) at a scan rate of 50 mV s^{-1}. (**b**) the analysis was performed using tetrabutylammonium tetrafluoroborate (0.1 M) as electrolyte in acetonitrile at a scan rate of 50 mV s^{-1}. The potential was determined versus Ag/AgCl electrode used as electrode of reference. Source data were provided as a Source Data file.

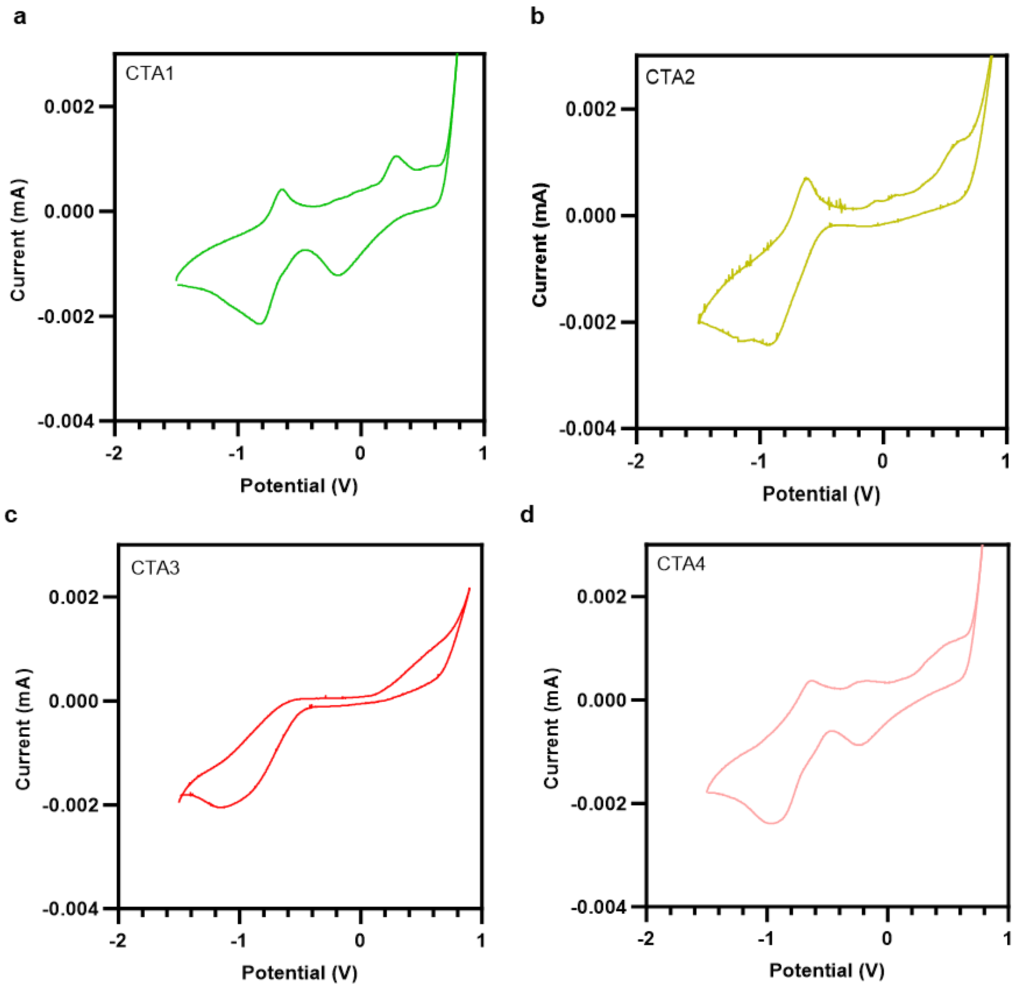

Supplementary Fig. 3 Cyclic voltammetry reduction peaks for different thiocarbonylthio RAFT agents. Note: the analysis was performed using tetrabutylammonium tetrafluoroborate (0.1 M) as electrolyte in acetonitrile ([CTA] = 10^{-3} M) at a scan rate of 50 mV s^{-1}. The potential was determined versus Ag/AgCl electrode used as electrode of reference and the reduction potential of thiocarbonylthio RAFT agent in PBK solution was adjusted by referencing the redox potential difference of riboflavin in PBK and acetonitrile solutions. Source data were provided as a Source Data file.

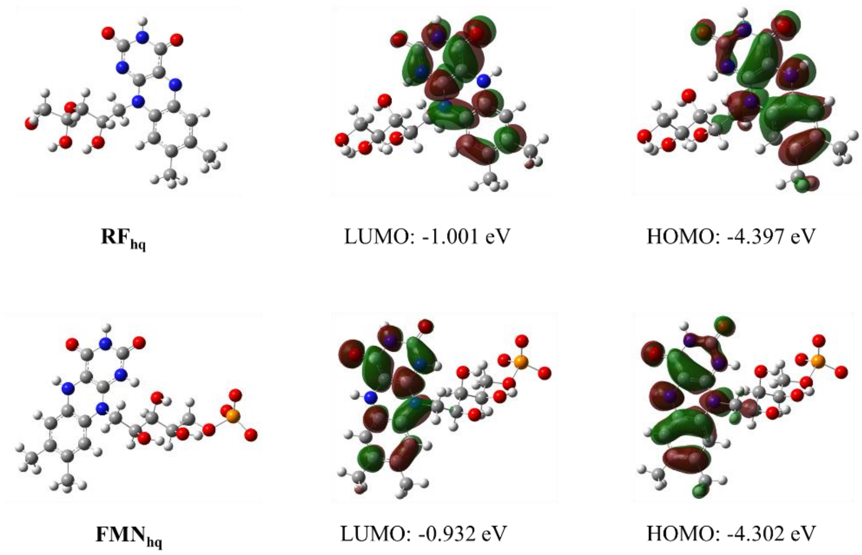

Supplementary Fig. 4 Calculated HOMO and LUMO topologies of FL_{hq}.

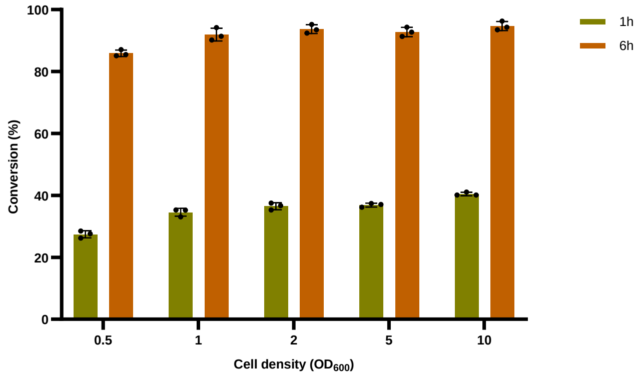

Supplementary Fig. 5 Monomer conversion of microorganism-triggered RAFT polymerization mediated by different concentrations of *S. oneidensis* after 1 h and 6 h of reaction time. Data were shown as the mean ± SD (n = 3). Source data were provided as a Source Data file.

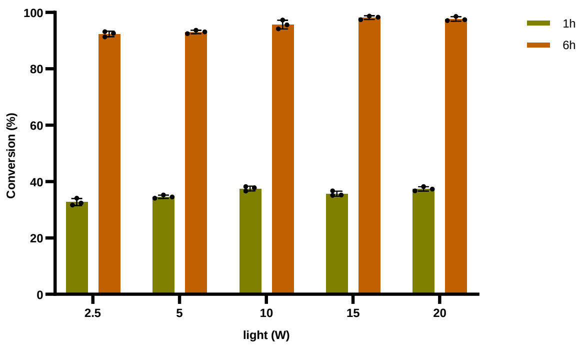

Supplementary Fig. 6 Monomer conversion of the *S. oneidensis*-triggered RAFT polymerization irradiated with different light intensity of blue LED light after 1 h and 6 h of reaction time. Data were shown as the mean ± SD (n = 3). Source data were provided as a Source Data file.

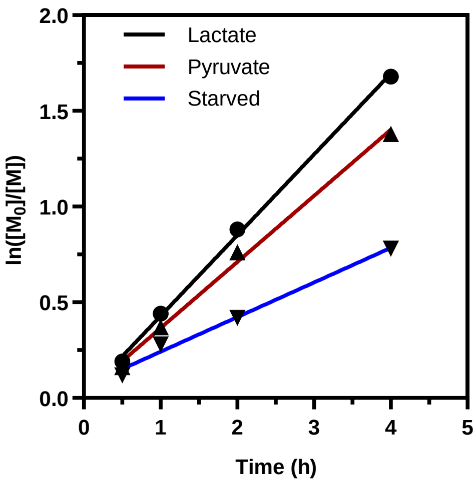

Supplementary Fig. 7 Kinetics of the *S. oneidensis*-triggered polymerization supplied with different carbon sources. Source data were provided as a Source Data file.

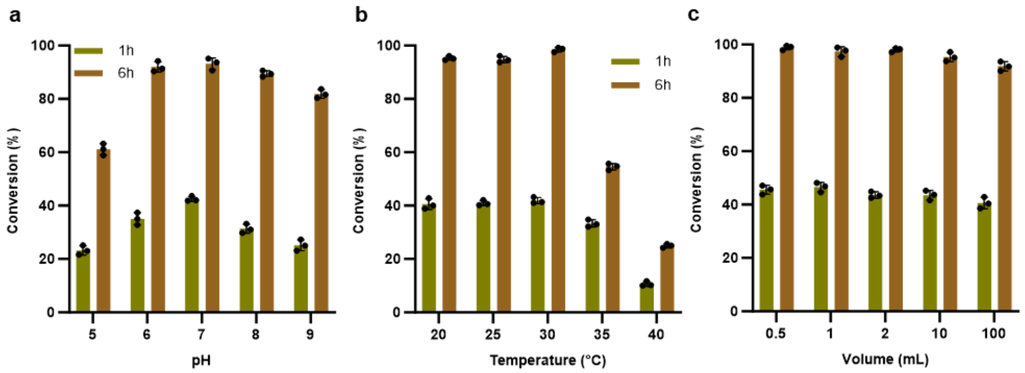

Supplementary Fig. 8 Stability of the polymerization system under different conditions. Monomer conversion of the *S. oneidensis*-triggered RAFT polymerization at different pH (**a**), temperature (**b**), in different system size (**c**), after 1 h and 6 h of reaction time. Data were shown as the mean ± SD (n = 3). Source data were provided as a Source Data file.

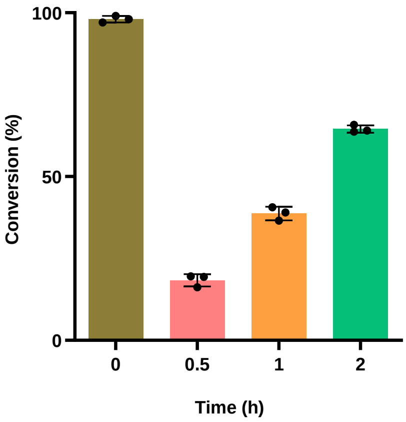

Supplementary Fig. 9 Monomer conversion ratio of time-staged cell-killing experiment at different time points (0.5 h, 1 h, and 2 h) in the course of the polymerization reaction. Data were shown as the mean ± SD (n = 3). Source data were provided as a Source Data file.

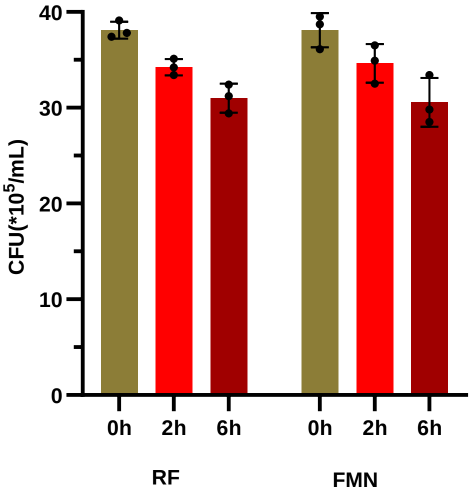

Supplementary Fig. 10 Residual microbial viability of *S. oneidensis* after participation in polymerization for 2 h and 6 h. Data showed mean ± SD of three independent experiments. Data were shown as the mean ± SD (n = 3). Source data were provided as a Source Data file.

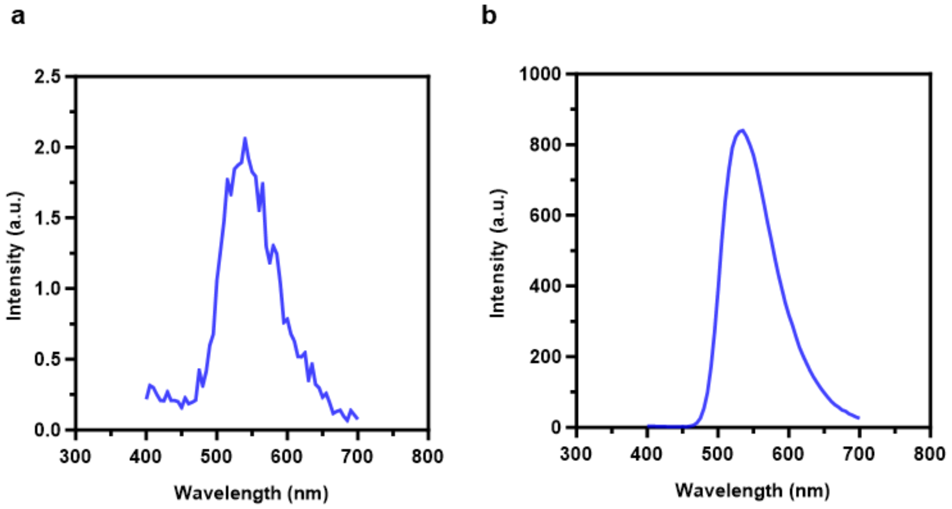

Supplementary Fig. 11 Fluorescence spectrum of RF_{hq} (**a**) and RF (**b**) in PBK (100 mM, pH7.5). [flavin] = 8 μM. Source data were provided as a Source Data file.

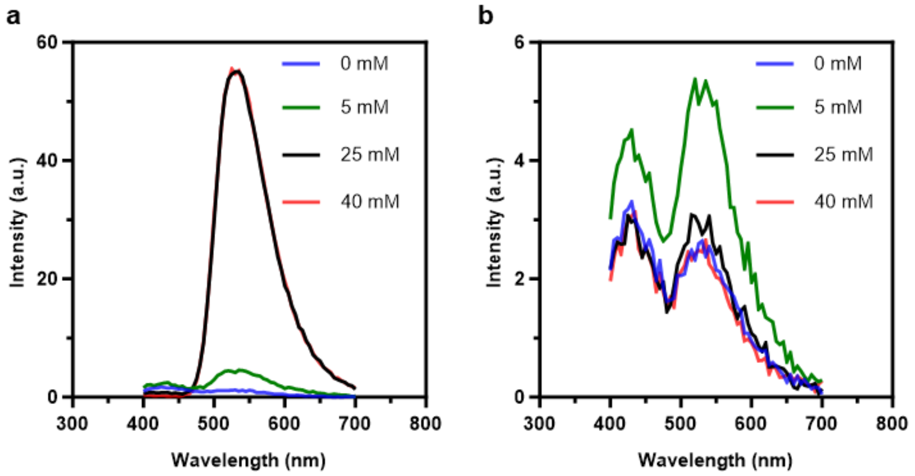

Supplementary Fig. 12 Fluorescence quenching emission spectrum of RF_{hq} with the quenching agent of different concentrations, CTA1 (**a**) and DMA (**b**). $[RF_{hq}]$ = 8 μM. Source data were provided as a Source Data file.

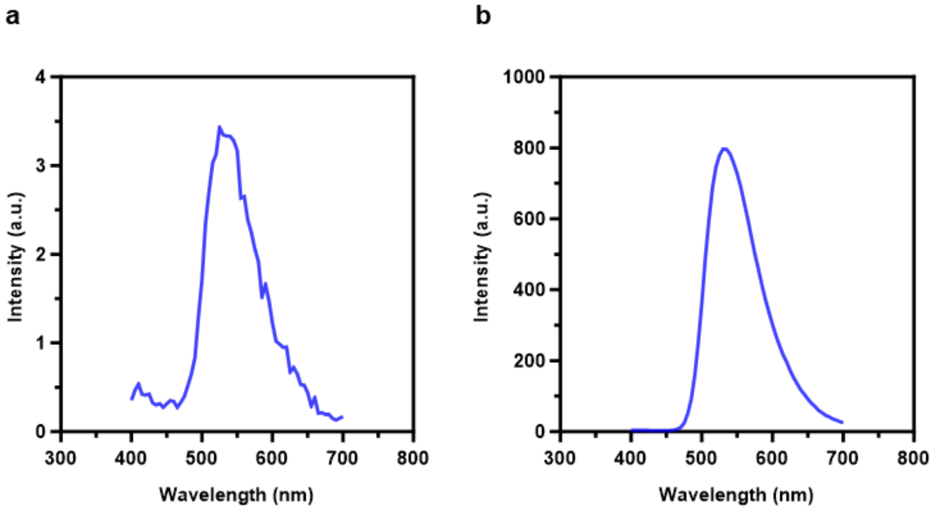

Supplementary Fig. 13 Fluorescence spectrum of FMN_{hq} (**a**) and FMN (**b**) in PBK (100 mM, pH7.5). $[FMN_{hq}]$ = 8 μM. Source data were provided as a Source Data file.

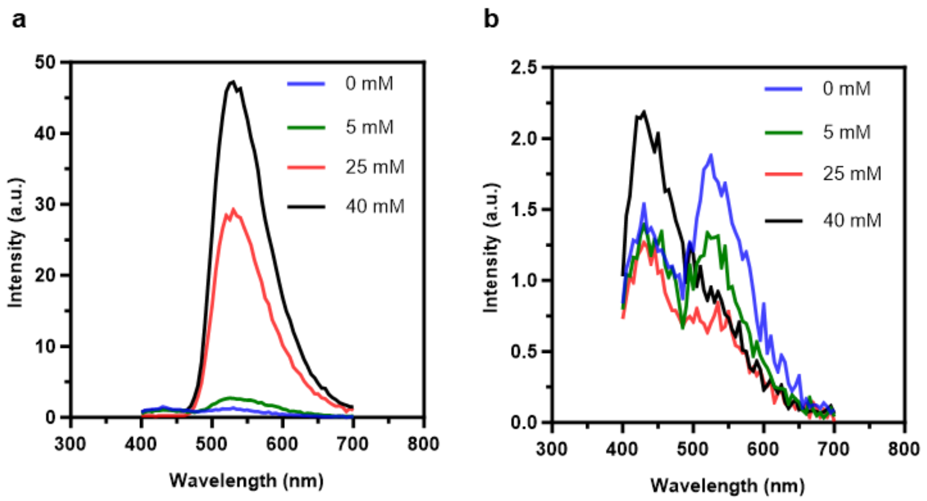

Supplementary Fig. 14 Fluorescence quenching emission spectrum of FMN_{hq} with the quenching agent of different concentrations, CTA1 (**a**) and DMA (**b**). [FMN_{hq}] = 8 μM. Source data were provided as a Source Data file.

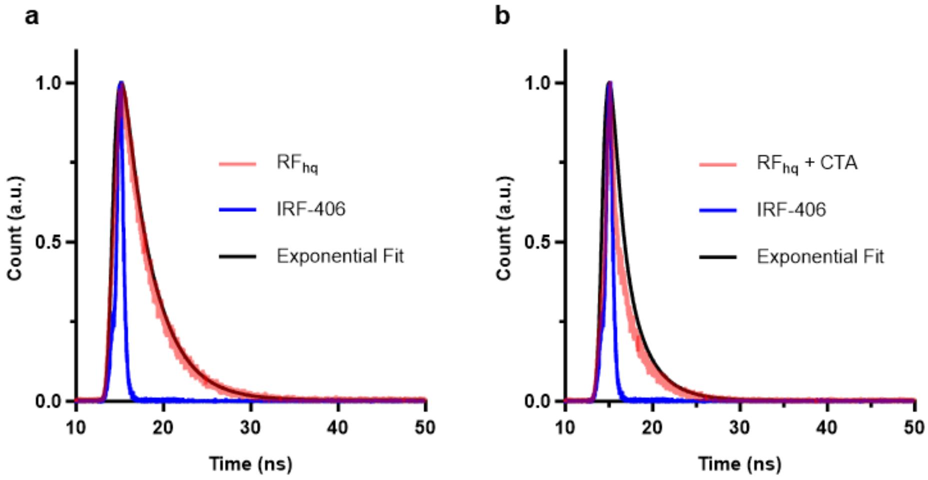

Supplementary Fig. 15 Time-resolved emission decay curves of RF_{hq} in the absence (**a**) and presence (**b**) of CTA1. The transient photoluminescence measurement was conducted on a spectrometer (HiLight 990, Oriental Spectra) equiped with a time correlated single photon counting (TCSPC) module (HiTime 990, Oriental Spectra). A picosecond 405 nm laser (pulse width < 250 ps, PLD405, Oriental Spectra) was used for optical excitation. The fluorescence emission wavelength was 528 nm. Source data were provided as a Source Data file.

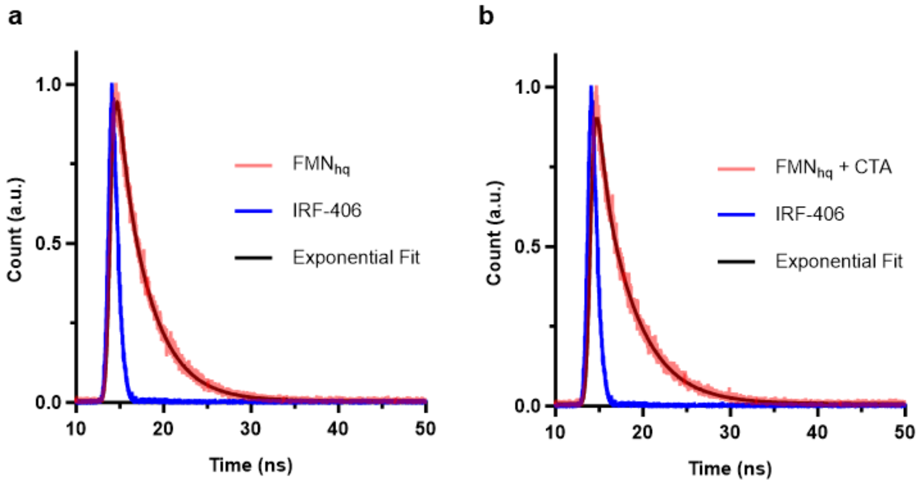

Supplementary Fig. 16 Time-resolved emission decay curves of FMN_{hq} in the absence (**a**) and presence (**b**) of CTA1. The transient photoluminescence measurement was conducted on a spectrometer (HiLight 990, Oriental Spectra) equiped with a time correlated single photon counting (TCSPC) module (HiTime 990, Oriental Spectra). A picosecond 405 nm laser (pulse width < 250 ps, PLD405, Oriental Spectra) was used for optical excitation. The fluorescence emission wavelength was 528 nm. Source data were provided as a Source Data file.

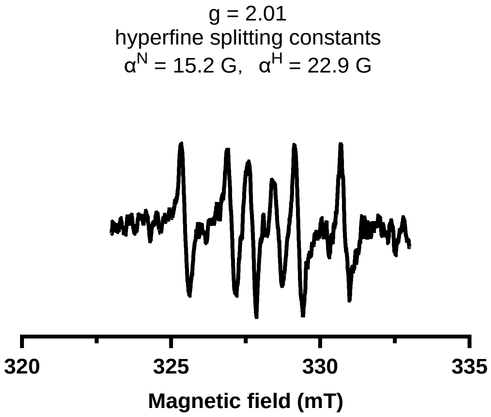

Supplementary Fig. 17 ESR spectra of riboflavin mediated RAFT polymerization. Source data were provided as a Source Data file.

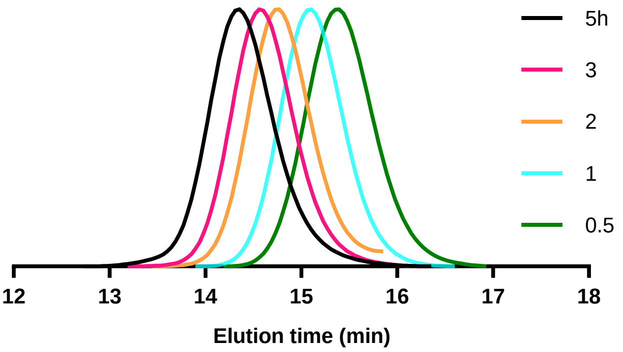

Supplementary Fig. 18 GPC traces of the *S. oneidensis*-triggered RAFT polymerization for light on-off experiment. Source data were provided as a Source Data file.

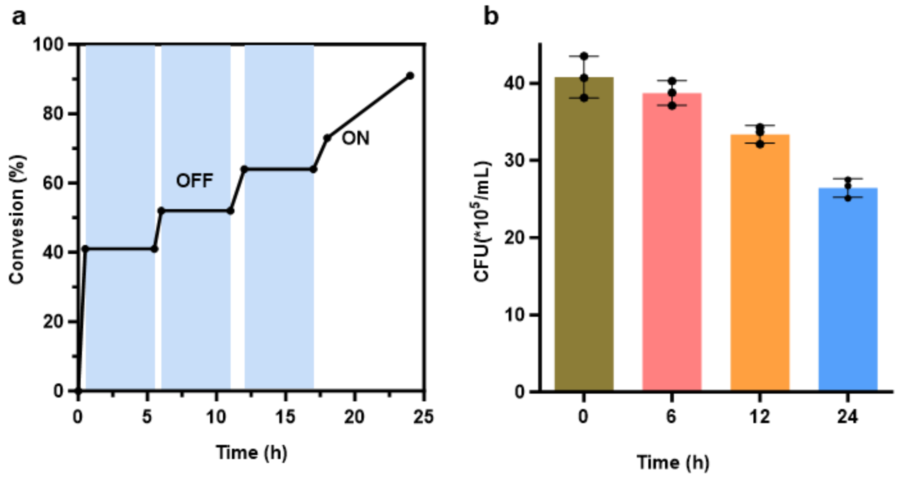

Supplementary Fig. 19 (a) Temporal control of the *S. oneidensis*-triggered RAFT polymerization with intermittent light for a long-term reaction. **(b)** Residual microbial viability of *S. oneidensis* after participation in microorganism-activated polymerization for a long-term reaction. Data in (**b**) are shown as the mean ± SD (n = 3). Source data were provided as a Source Data file.

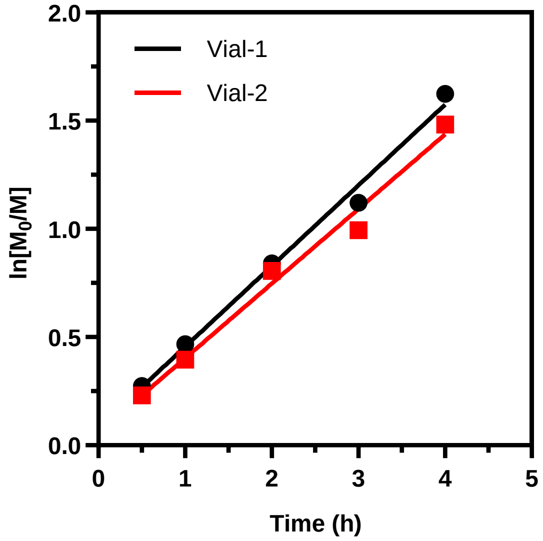

Supplementary Fig. 20 Kinetics of the *S. oneidensis*-triggered RAFT polymerization under anaerobic (Vial-1) and aerobic (Vial-2) conditions. Source data were provided as a Source Data file.

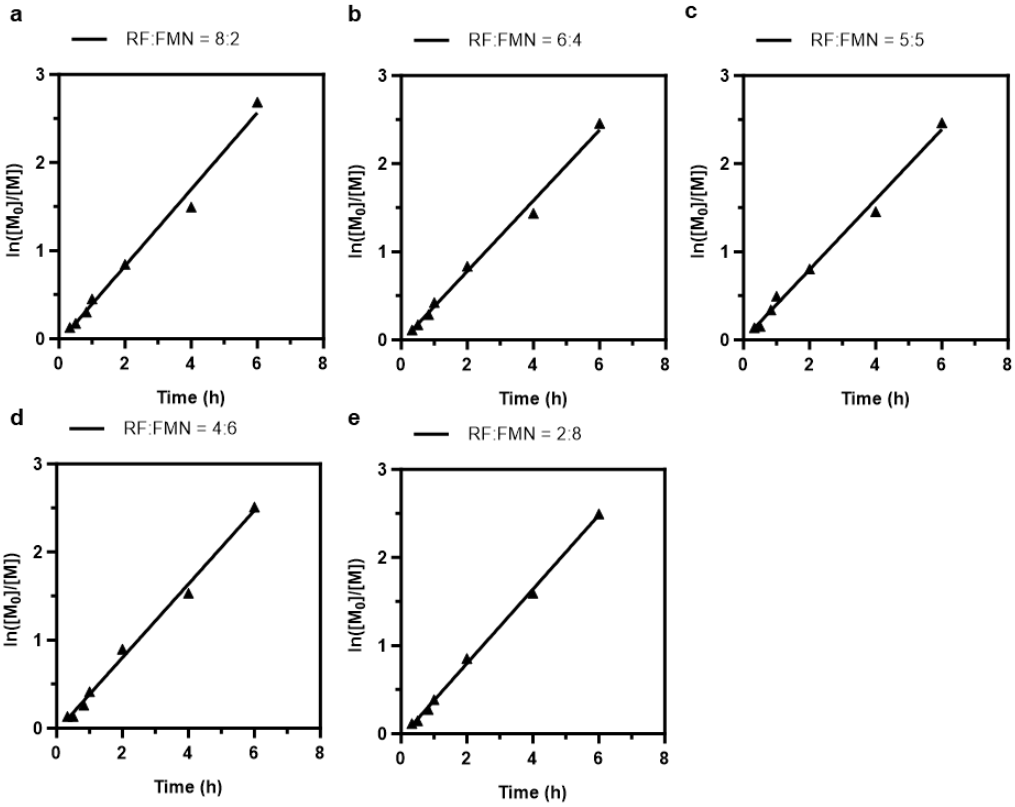

Supplementary Fig. 21 Kinetic results of the *S. oneidensis*-triggered RAFT polymerization mediated by the flavin mixtures with different ratios. Source data were provided as a Source Data file.

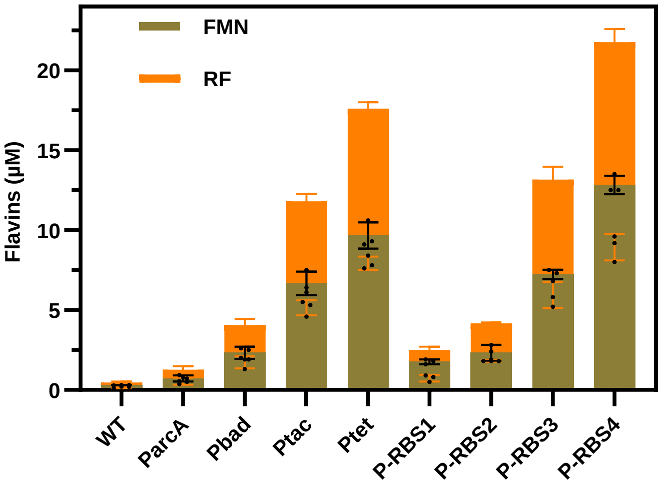

Supplementary Fig. 22 Quantification of flavins (FMN and RF) produced by genetically engineered *S. oneidensis* harboring different promotors and RBS, respectively. Data are shown as the mean ± SD (n = 3). Source data were provided as a Source Data file.

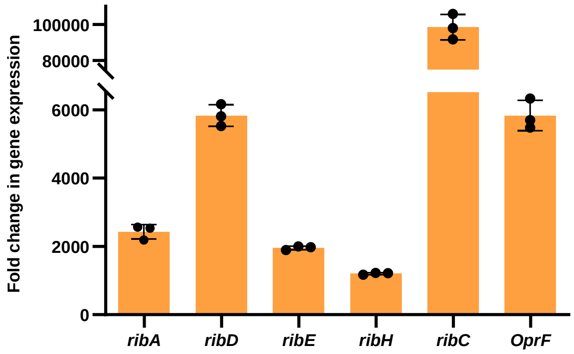

Supplementary Fig. 23 The fold changes (strain P-RBS4 *vs* WT) of the expression level of the genes *ribA*, *ribD*, *ribE*, *ribH*, *ribC*, and *oprF* in the engineered *S. oneidensis* P-RBS4 and the wild-type *S. oneidensis* MR-1. Real-time quantitative reverse transcription PCR (RT-qPCR) analysis was performed by using Power Up SYBR Green Master Mix (Thermo Fisher, A25742, USA). The gene *gyrB*, which encodes DNA gyrase, was selected as the internal reference gene due to its consistently stable expression across various growth phases [1]. The primers used for RT-qPCR were listed in Supplementary Table 12. The fold change (FC) in gene expression was calculated using the equation [2] $FC = 2^{-(\Delta C_{T,P-RBS4} - \Delta C_{T,WT})}$, where ΔC_T is equal to the difference in threshold cycles for target and internal reference ($\Delta C_{T,X}$ - $\Delta C_{T,R}$), and C_T is the threshold cycle for reference amplification. Data were shown as the mean ± SD (n = 3). Source data were provided as a Source Data file.

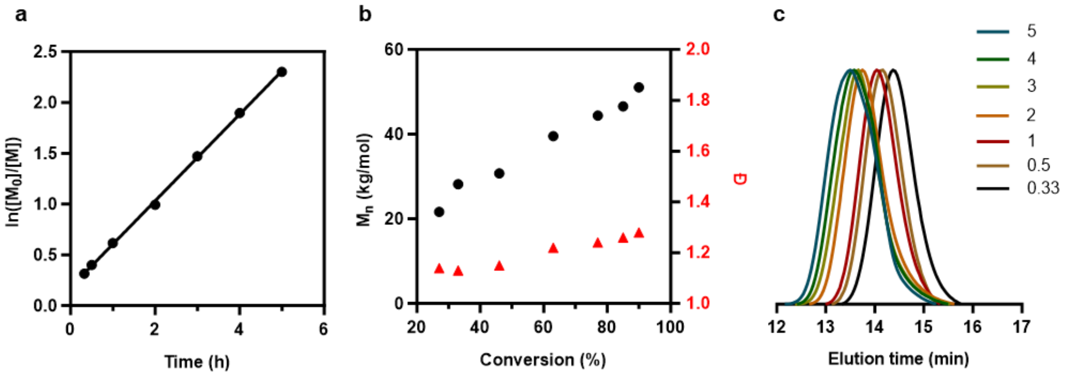

Supplementary Fig. 24 Kinetic results of the *S. oneidensis*-triggered RAFT polymerization mediated by the engineered *S. oneidensis* strain P-RBS4. (**a**) First-order polymerization kinetics of P-RBS4-triggered RAFT. (**b**) Molecular weight (black circles) and dispersity (red squares) evolution. (**c**) GPC traces of the polymerizations. Source data were provided as a Source Data file.

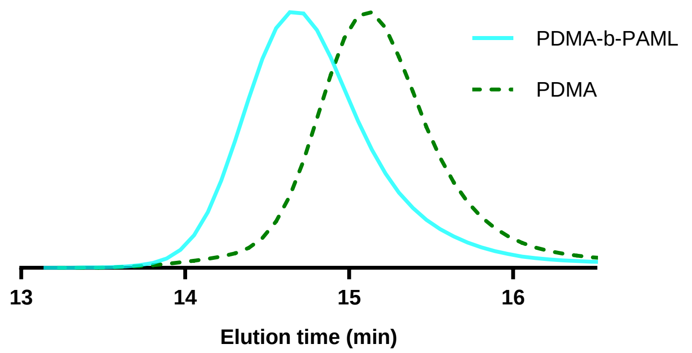

Supplementary Fig. 25 GPC traces of diblock polymer PDMA-b-PAML with CTA1. Source data were provided as a Source Data file.

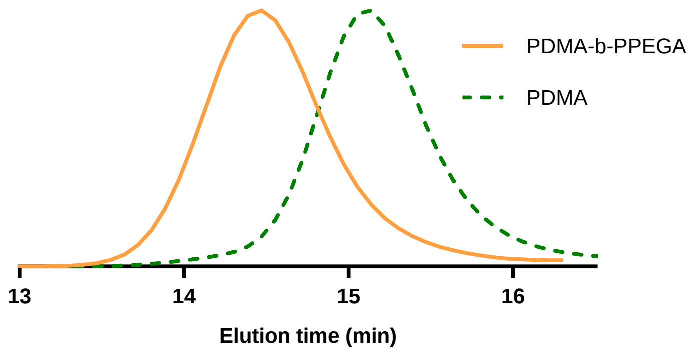

Supplementary Fig. 26 GPC traces of diblock polymer PDMA-b-PPPEGA with CTA1. Source data were provided as a Source Data file.

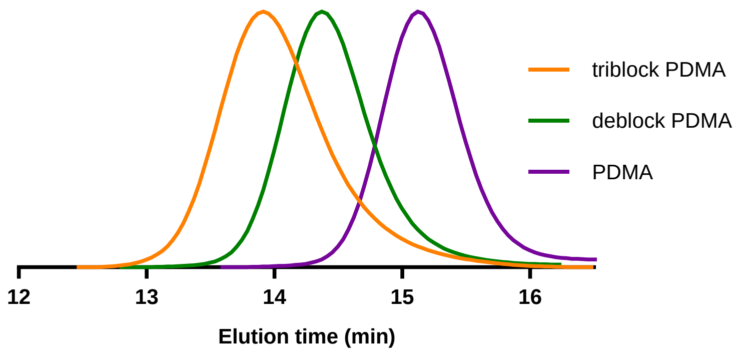

Supplementary Fig. 27 GPC traces of diblock polymer PDMA-b-PDMA and triblock polymer PDMA-b-PDMA-b-PDMA with CTA2. Source data were provided as a Source Data file.

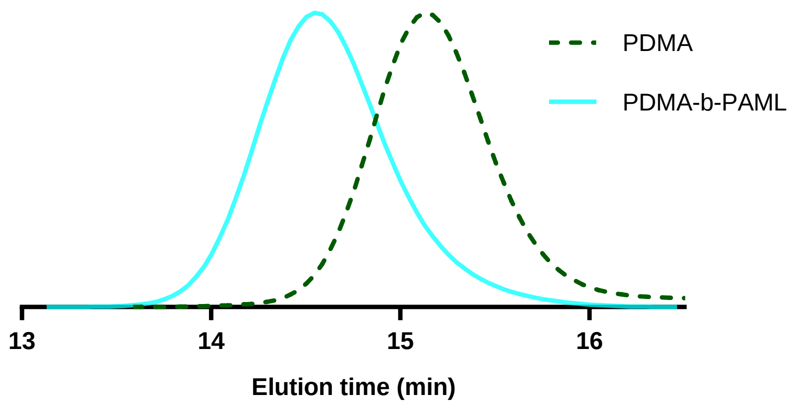

Supplementary Fig. 28 GPC traces of diblock polymer PDMA-b-PAML with CTA2. Source data were provided as a Source Data file.

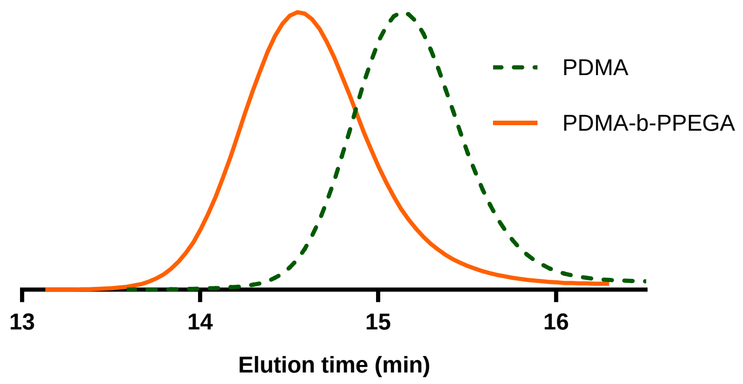

Supplementary Fig. 29 GPC traces of diblock polymer PDMA-b-PPPEGA with CTA2. Source data were provided as a Source Data file.

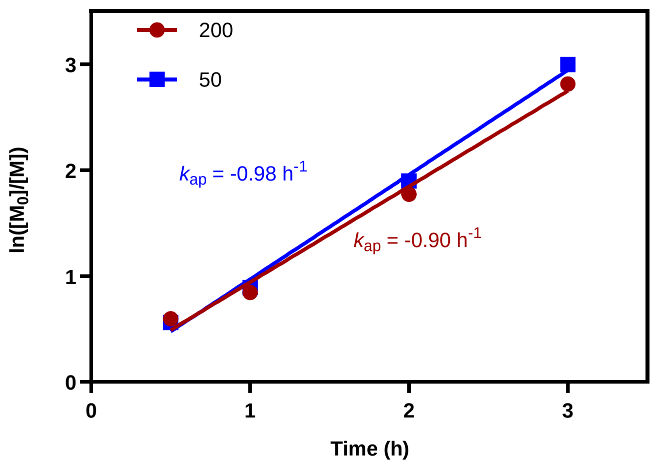

Supplementary Fig. 30 Polymerization kinetics of the *S. oneidensis*-triggered RAFT polymerization performed in the 96 and 384-well plates. Polymerization conditions: [riboflavin] = 10 μM, *S. oneidensis* [OD_{600}] = 1, [DMA]= 1 M, blue LED light (10 W, 460 nm), room temperature. Source data were provided as a Source Data file.

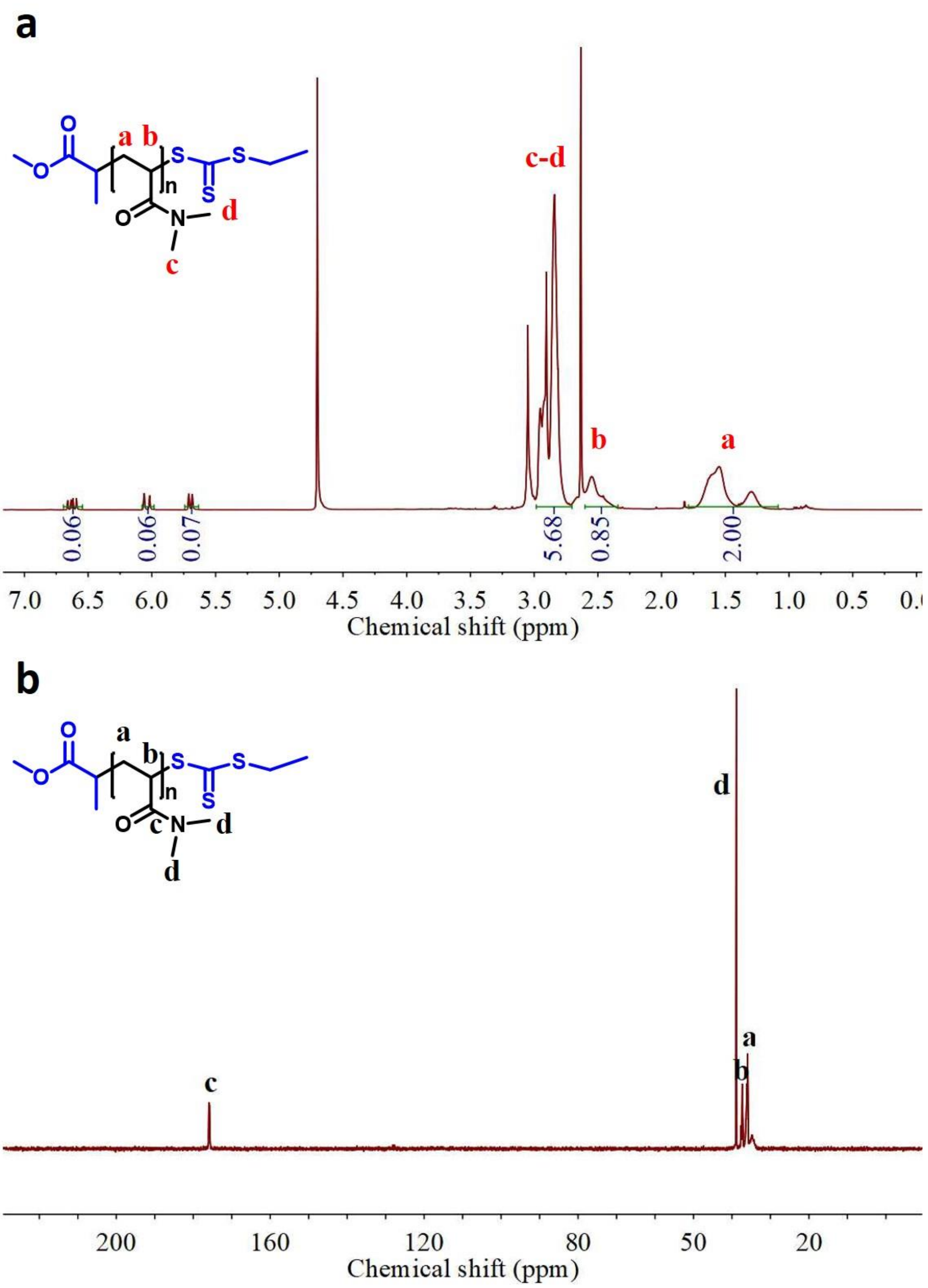

Supplementary Fig. 31 NMR spectra of PDMA. (**a**) ^{1}H NMR in D_2O. (**b**) ^{13}C NMR in D_2O.

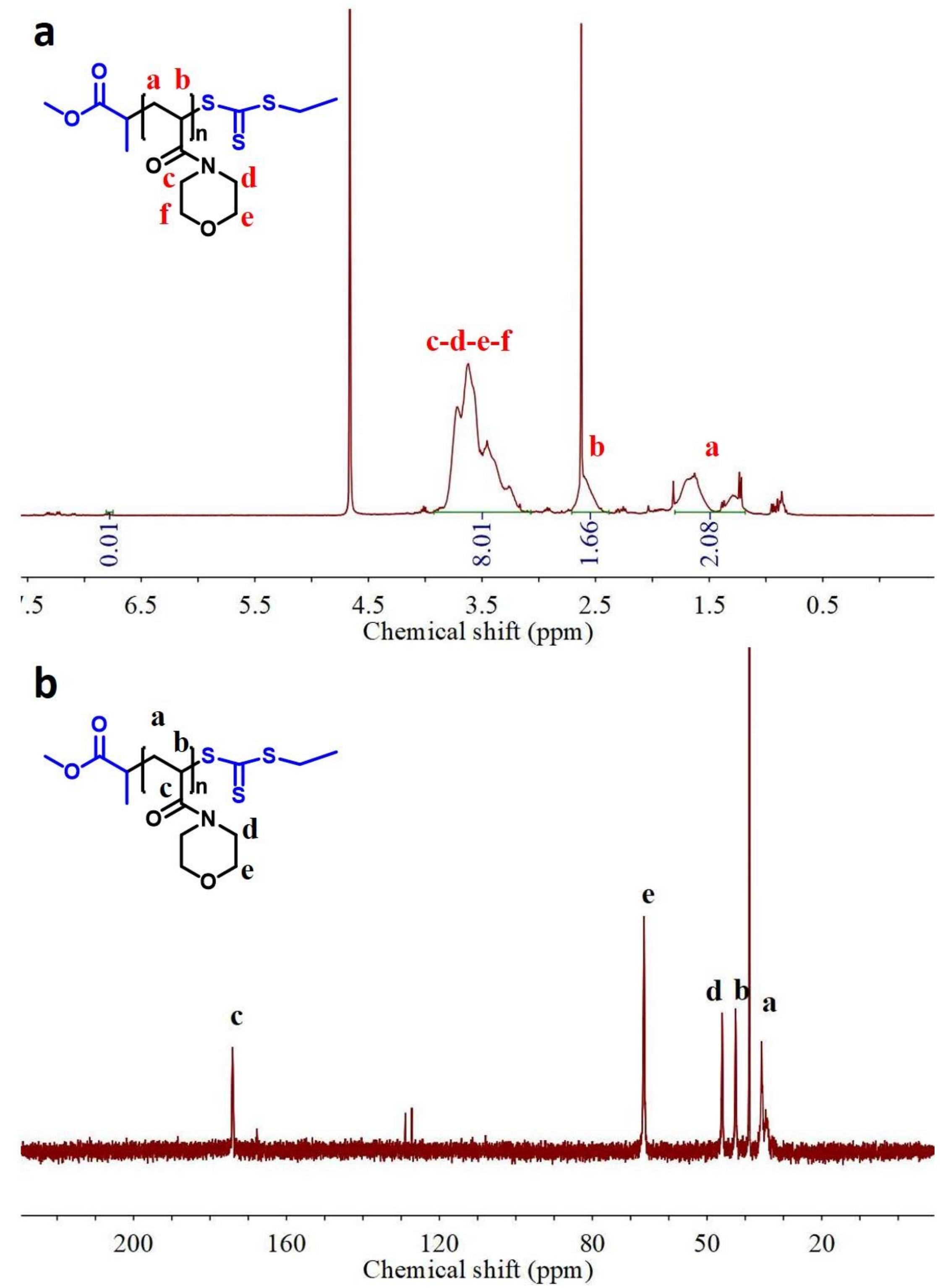

Supplementary Fig. 32 NMR spectra of PAML. (**a**) ^{1}H NMR in D_2O. (**b**) ^{13}C NMR in D_2O.

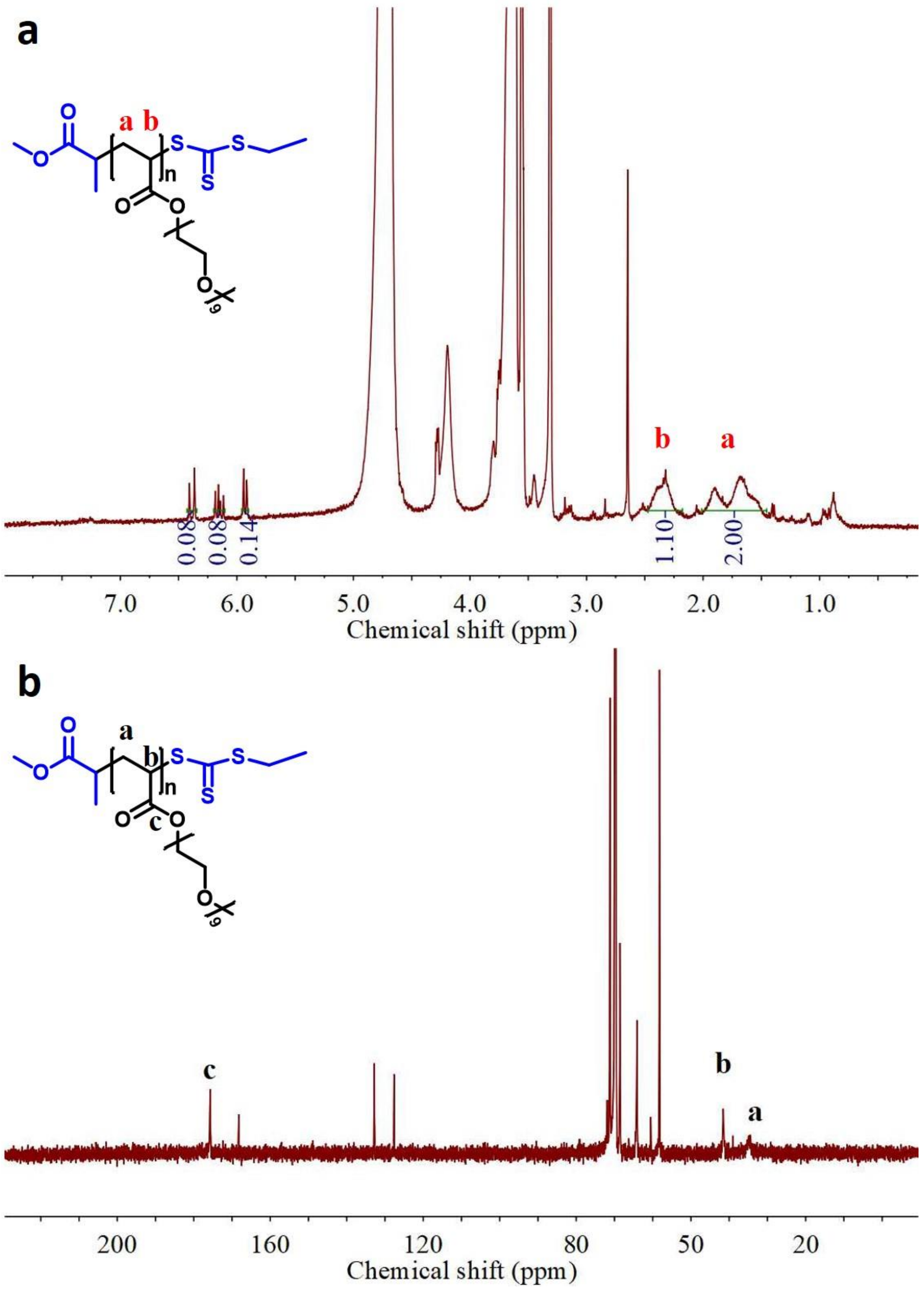

Supplementary Fig. 33 ^{1}H NMR spectra of PPEGA in D_2O. (**a**) ^{1}H NMR in D_2O. (**b**) ^{13}C NMR in D_2O.

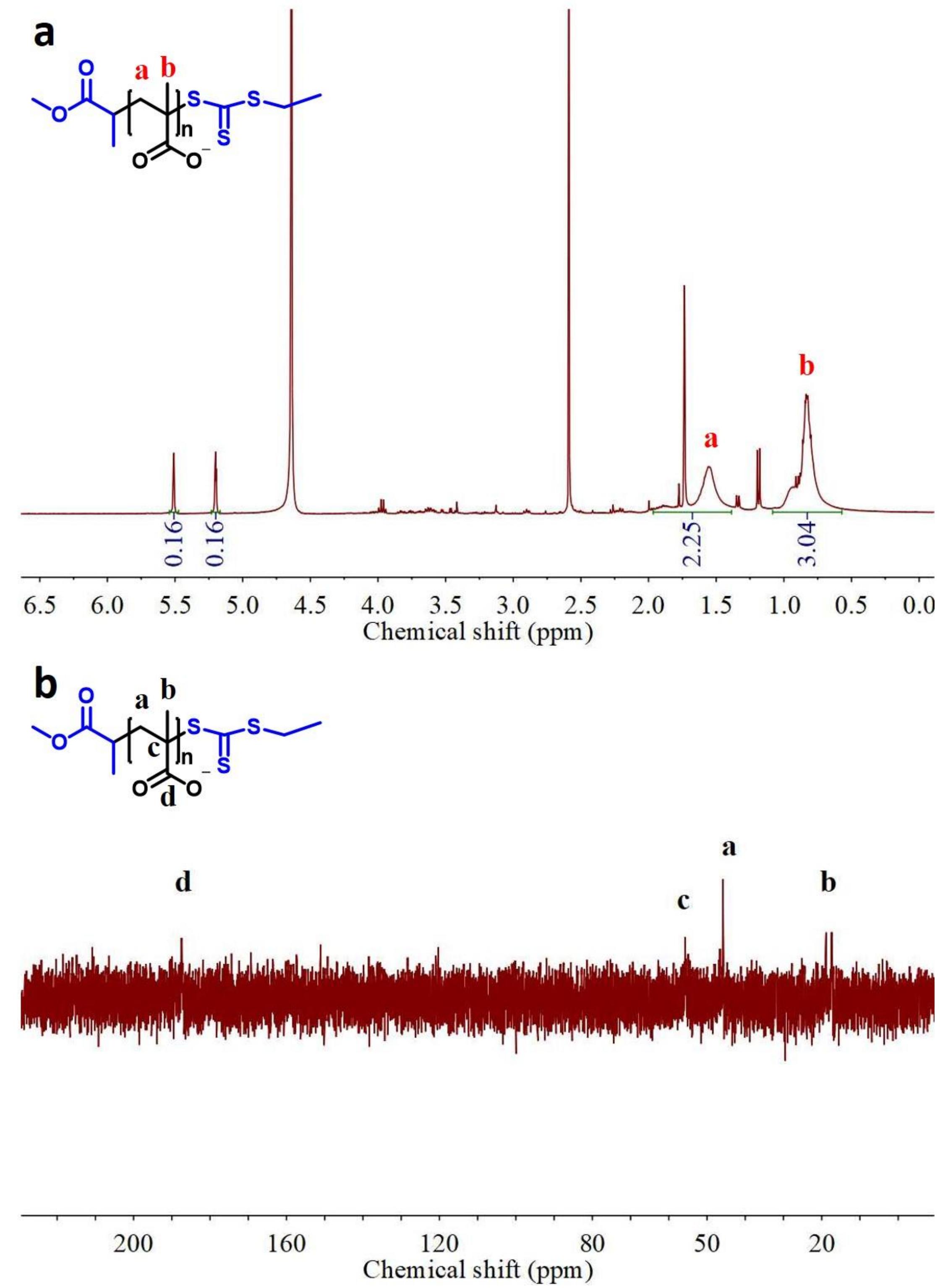

Supplementary Fig. 34 NMR spectra of PMAA. (**a**) ^{1}H NMR in D_2O. (**b**) ^{13}C NMR in D_2O.

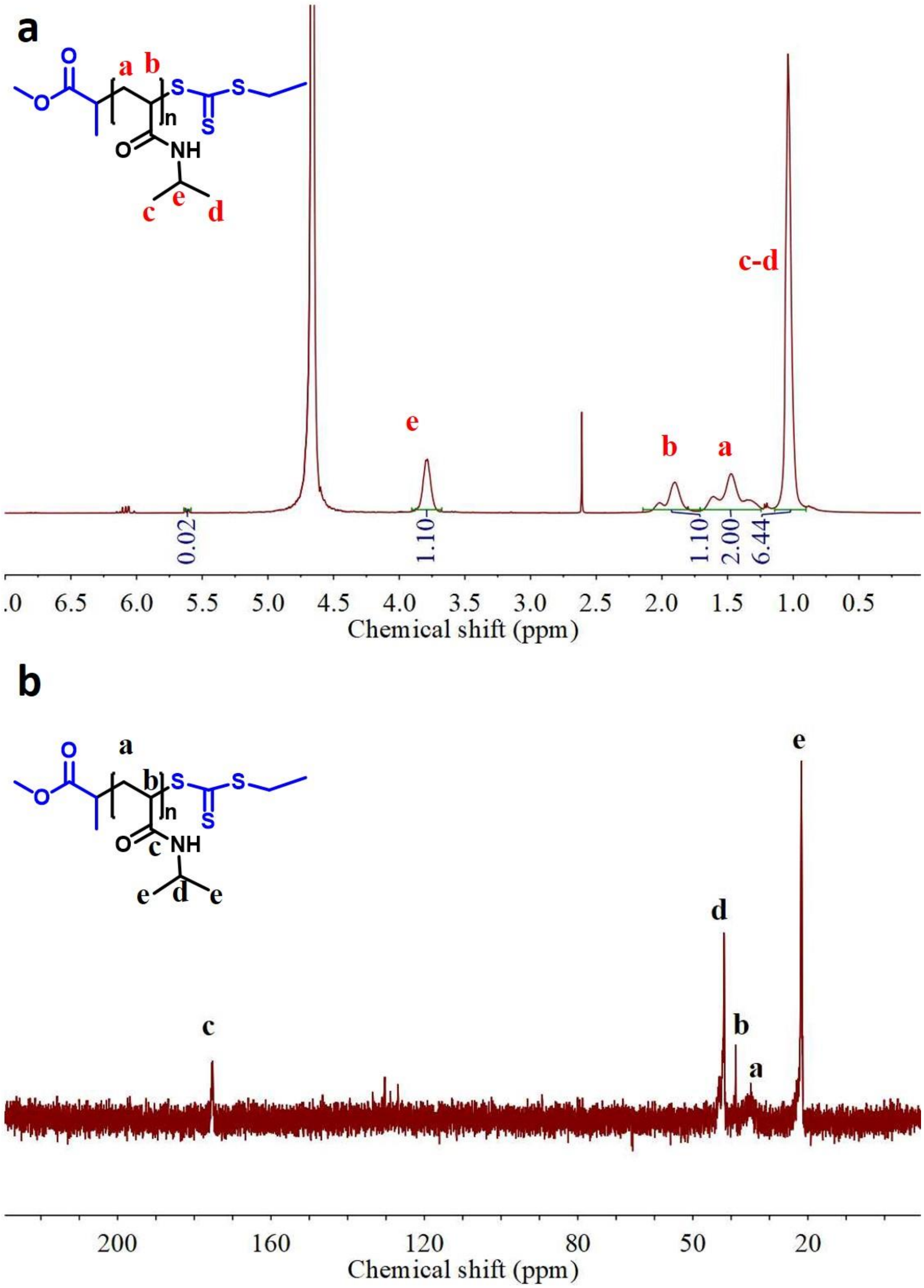

Supplementary Fig. 35 NMR spectra of PNIPAM. (**a**) ^{1}H NMR in D_2O. (**b**) ^{13}C NMR in D_2O.

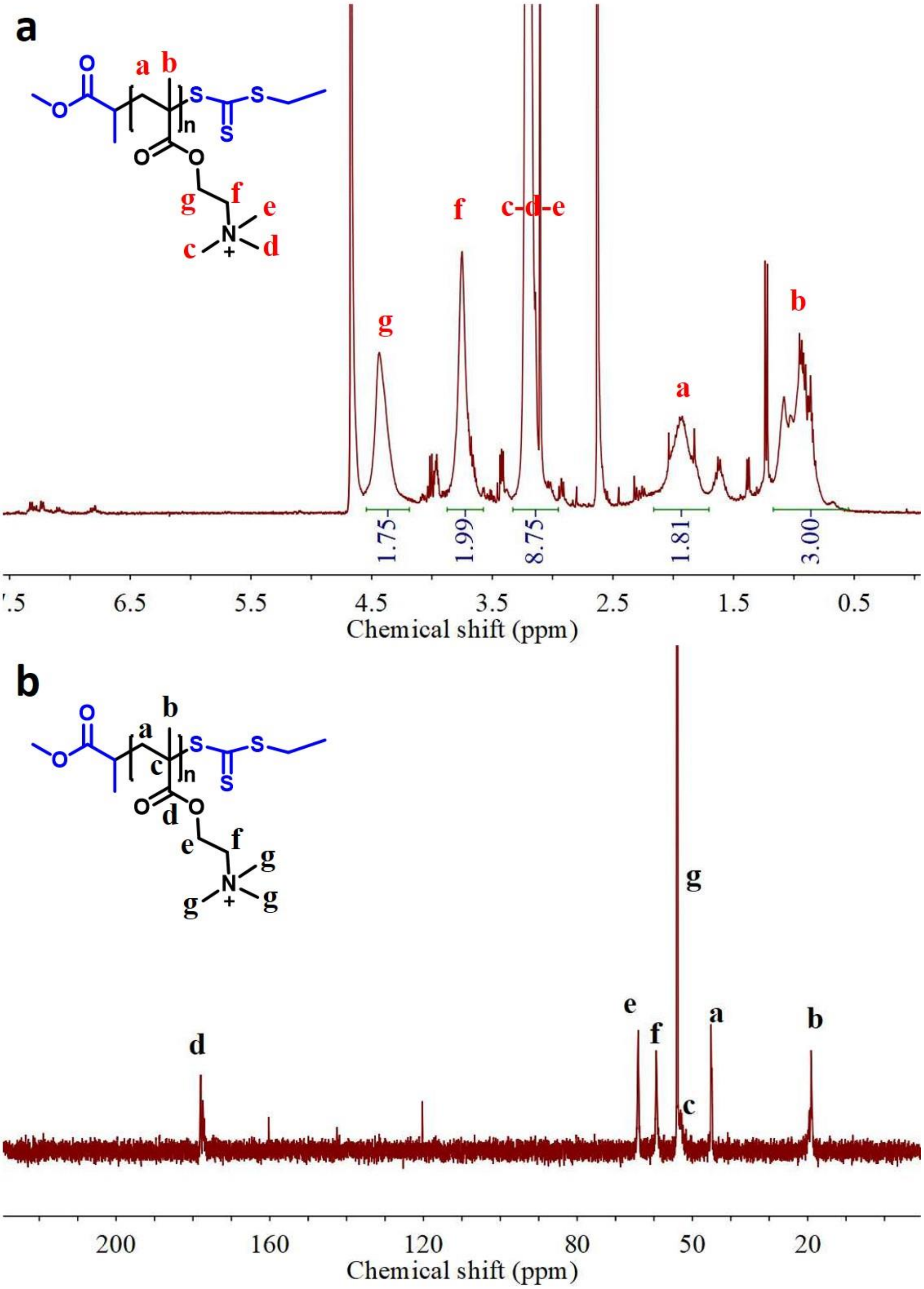

Supplementary Fig. 36 NMR spectra of PTMAEMA. (**a**) ^{1}H NMR in D_2O. (**b**) ^{13}C NMR in D_2O.

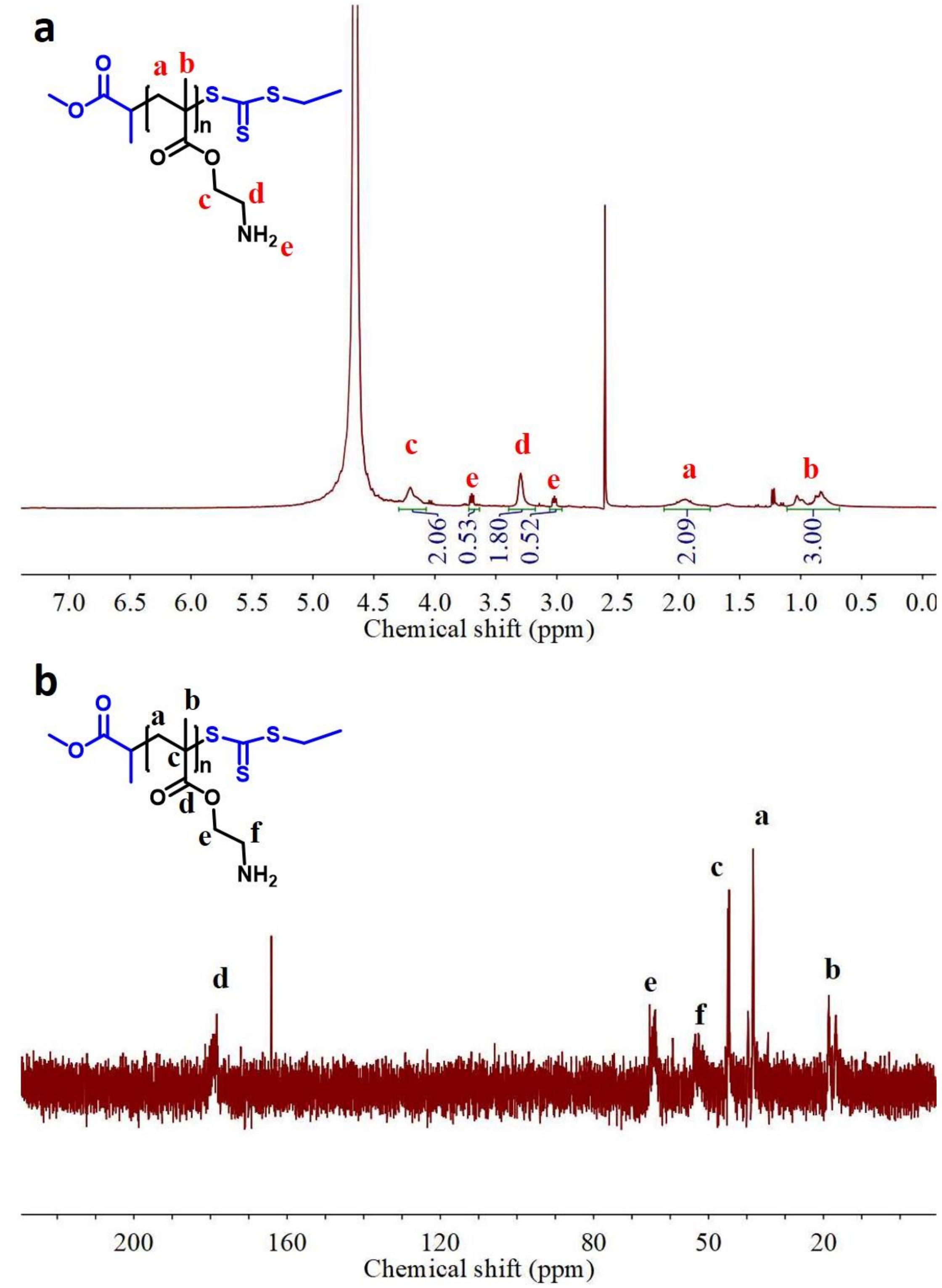

Supplementary Fig. 37 NMR spectra of PAOMA. (**a**) ^{1}H NMR in D_2O. (**b**) ^{13}C NMR in D_2O.

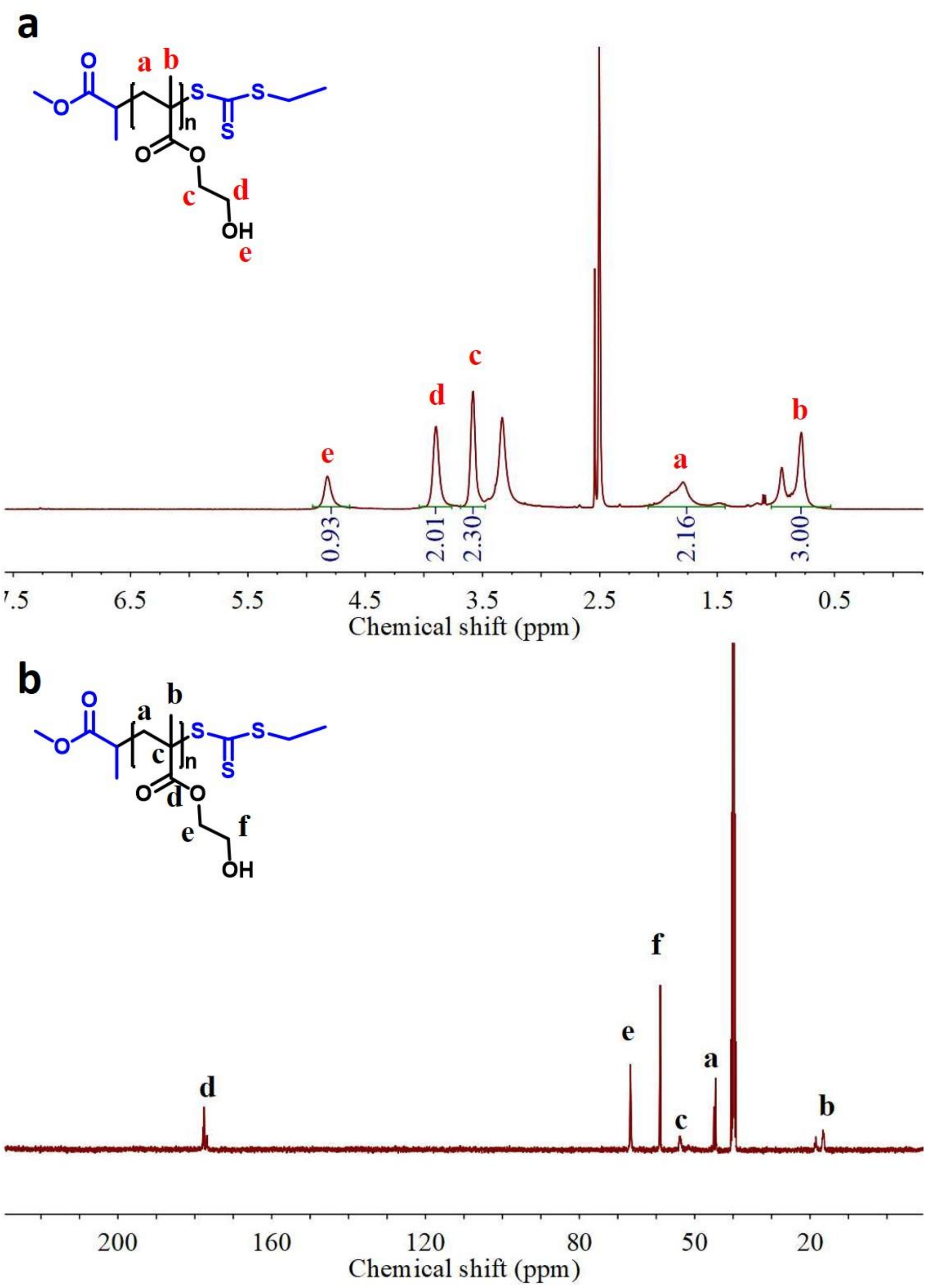

Supplementary Fig. 38 NMR spectra of PHEMA. (**a**) ^{1}H NMR in DMSO-d6. (**b**) ^{13}C NMR in DMSO-d6.

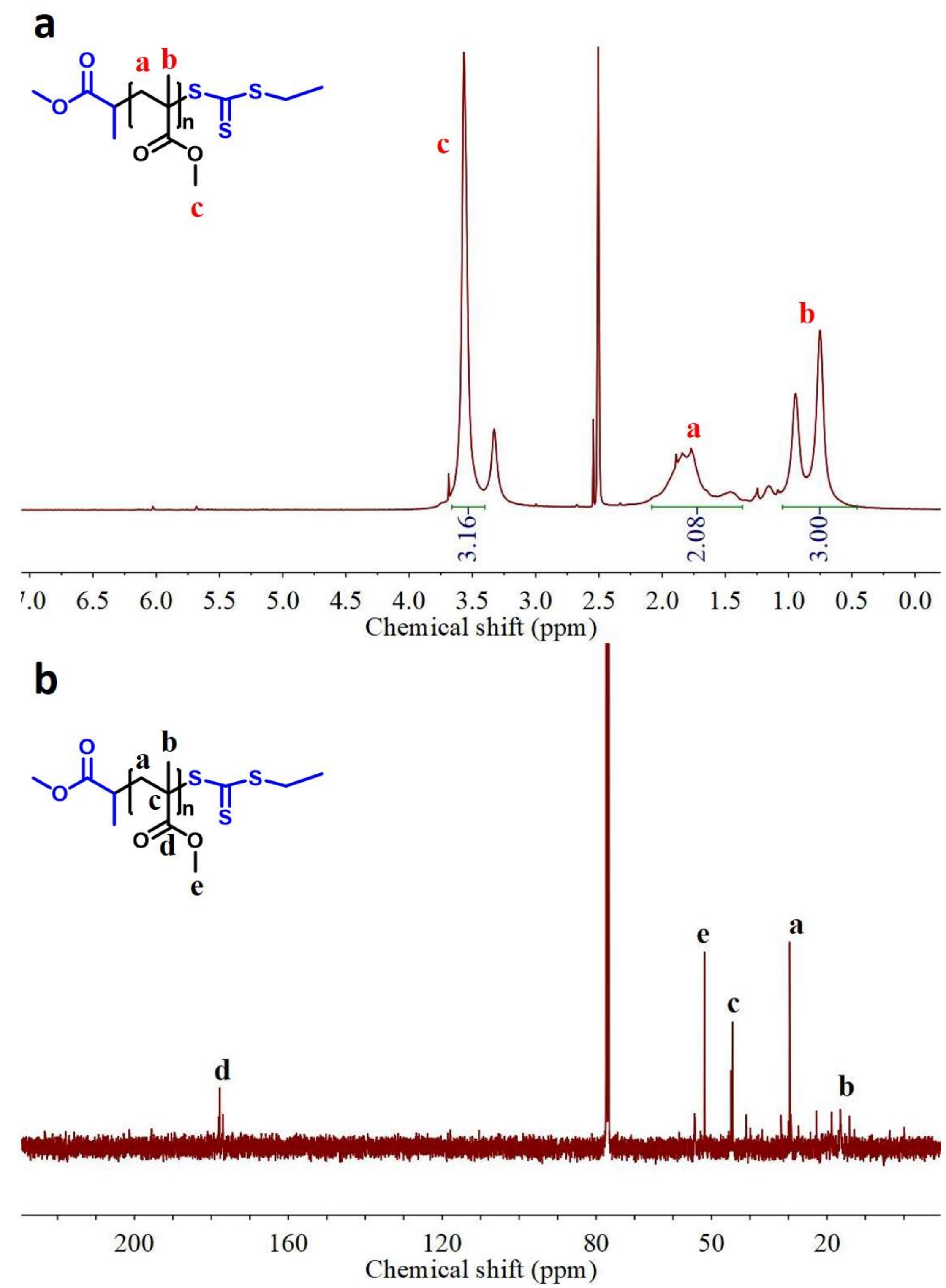

Supplementary Fig. 39 NMR spectra of PMMA. (**a**) ^{1}H NMR in DMSO-d6. (**b**) ^{13}C NMR in CDCl3.

Supplementary Table 1 Electronic properties and Gibbs energies of electron transfer (ΔG_{EET}) from RF_{hq} to CTA.

E_{red} (RF_{hq}) (V vs. Ag/AgCl)	E_{red} (FMN_{hq}) (V vs. Ag/AgCl)[3]	E_{red} (CTA) (V vs. Ag/AgCl)		ΔG_{EET} for reduction of CTA (eV)[a]
-0.45	-0.49	CTA1	-0.70	>0
		CTA 2	-0.99	>0
		CTA 3	-0.83	>0
		CTA 4	-0.84	>0

[a]These values were estimated as follows equation: $\Delta G_{EET} = e\,[E_{red}(RF_{hq}) - E_{red}(CTA)]$ [4].

Supplementary Table 2 Free energy value, and the energy used for reaction(a.u.).

RF	RF_{hq}	RF_{hq}*	RF_{sq}	CTA1	$CTA1^-$
-1329.5316	-1330.7299	-1330.6575	-1330.6203	-1618.6521	-1618.7645

$CTA1\text{-}R^{\cdot}$	$CTA1\text{-}Z^-$	FMN	FMN_{hq}	FMN_{hq}*	FMN_{sq}
-306.8876	-1311.9139	-1896.1244	-1897.3244	-1897.2553	-1897.2141

Supplementary Table 3 Free energy change of system RF and FMN (kcal/mol).

System	State_0	State_1	State_2	State_3	State_4
RF	0	-13.44	31.98	-1.788	-24.98
FMN	0	-14.47	28.84	-1.39	-24.59

Supplementary Table 4 Electronic properties and Gibbs energies of electron transfer (ΔG_{PET}) from excited RF_{hq}* to CTA.

E_{red}^*(RF_{hq}) (V vs. Ag/AgCl)	E_{red}^*(FMN_{hq}) (V vs. Ag/AgCl)[3]	E_{red} (CTA) (V vs. Ag/AgCl)		ΔG_{PET} for reduction of CTA (eV) [a]
-2.52	-2.30	CTA1	-0.70	<0
		CTA 2	-0.99	<0
		CTA 3	-0.83	<0
		CTA 4	-0.84	<0

[a]These values were estimated as follows equation: $\Delta G_{PET} = e\,[E_{red}^*(RF_{hq}) - E_{red}(CTA)]$ [4].

Supplementary Table 5 The *S. oneidensis*-triggered polymerization of DMA under varied conditions.

Entry	*S. oneidensis* (OD_{600})	DMA/CTA/Flavin	light	Conversion (%)	*Đ*
1	1	500/1/0.0025(RF)	ON	95	1.15
2	1	500/1/0.0025(FMN)	ON	90	1.14
3	0	500/1/0.0025	ON	<5	-
4	1	500/1/0	ON	<1	-
5	0	500/1/0.0025(RF_{hq})	ON	25	1.18
6	1	500/1/0.0025	OFF	<1	-
7	1	500/0/0.0025	ON	99	2.6

Conversion ratio was determined by ^{1}H NMR spectroscopy. Polydispersity (*Đ*) was determined by GPC analysis. Polymerization conditions: [DMA]= 1 M, blue LED light (10 W, 460 nm), room temperature, 6h, cultured anaerobically with magnetic stirring. Monomer conversion determined by ^{1}H NMR. Molecular dispersity determined by GPC.

Supplementary Table 6 Results of the *S. oneidensis*-triggered RAFT polymerization mediated by the wild-type *S. oneidensis* strain.

Entry	Time (h)	Conversion (%)	$M_{n,GPC}$ (kg mol^{-1})	$M_{n,th}$ (kg mol^{-1})	*Đ*
1	0.33	13	10.1	6.7	1.19
2	0.5	17	15.0	8.6	1.13
3	0.83	25	19.7	12.6	1.12
4	1	35	21.4	17.6	1.13
5	2	58	30.5	29.0	1.16
6	4	79	39.7	39.3	1.15
7	6	90	50.7	44.8	1.19

Conversion ratio was determined by ^{1}H NMR spectroscopy. Molecular weight (M_n) and polydispersity (*Đ*) were determined by GPC analysis. Polymerization conditions: [riboflavin] = 5 µM, *S. oneidensis* [OD_{600}] = 1, [DMA]= 1 M, [CTA] = 2 mM, blue LED light (10 W, 460 nm), room temperature, 6h, cultured anaerobically with magnetic stirring. Monomer conversion determined by ^{1}H NMR. Molecular dispersity determined by GPC.

Supplementary Table 7 Preparation of diverse homopolymers and block copolymers with CTA1 or CTA2.

Entry	CTA	Block polymer	Conversion (%)	Monomer /Macro	$M_{n,\,GPC}$ (kg mol^{-1})	$Đ$
1		PDMA macroinitiator			10.3	1.12
2		PDMA-b-PDMA	95	125/1	21.7	1.15
3	CTA1	PDMA-b-PDMA-b-PDMA	99	125/1	29.3	1.37
4		PDMA-*b*-PNAM	78	100/1	14.7	1.24
5		PDMA-*b*-PPEGA	94	28/1	18.5	1.20
6		PDMA macroinitiator			10.4	1.15
7		PDMA-*b*-PDMA	96	125/1	21.8	1.16
8	CTA 2	PDMA-*b*-PDMA-b-PDMA	99	125/1	30.6	1.25
		PDMA-*b*-PNAM	84	100/1	18.9	1.13
		PDMA-*b*-PPEGA	93	28/1	17.6	1.16

Conversion ratio was determined by ^{1}H NMR spectroscopy. Molecular weight (M_n) and polydispersity ($Đ$) were determined by GPC analysis.

Supplementary Table 8 Results of the *S. oneidensis*-triggered RAFT polymerization for light on-off experiment.

Entry	Light-Time (h)	Conversion (%)	$M_{n,GPC}$ (kg mol^{-1})	$M_{n,th}$ (kg mol^{-1})	$Đ$
1	0.5	38	8.9	9.6	1.12
2	1	53	11.7	13.4	1.10
3	2	68	15.9	17.1	1.11
4	3	81	18.6	20.3	1.11
5	5	98	22.6	24.5	1.15

Conversion ratio was determined by ^{1}H NMR spectroscopy. Molecular weight (M_n) and polydispersity ($Đ$) were determined by GPC analysis. Polymerization conditions: [riboflavin] = 5 μM, *S. oneidensis* [OD_{600}] = 1, [DMA]= 0.5 M, [CTA] = 2 mM, blue LED light (10 W, 460 nm), room temperature, 6h, cultured anaerobically with magnetic stirring. Monomer conversion determined by ^{1}H NMR. Molecular dispersity determined by GPC.

Supplementary Table 9 Strains and plasmids used in this study.

Strain or plasmid	Description	Source
S. oneidensis strains		
MR-1	Lake Oneida isolate	Our Lab
WT	MR-1 harboring pYYDT	Our Lab
P$_{\text{arcA}}$	MR-1 harboring pYYDT-P$_{\text{arcA}}$-*ribADEHC*	This study
P$_{\text{bad}}$	MR-1 harboring pYYDT-P$_{\text{bad}}$-*ribADEHC*	This study
P$_{\text{tac}}$	MR-1 harboring pYYDT-P$_{\text{tac}}$-*ribADEHC*	This study
P$_{\text{tet}}$	MR-1 harboring pYYDT-P$_{\text{tet}}$-*ribADEHC*	This study
P-RBS1	MR-1 harboring pYYDT-P$_{\text{tet}}$-*ribADEHC*-P$_{\text{tet}}$-RBS1-*OprF*	This study
P-RBS2	MR-1 harboring pYYDT-P$_{\text{tet}}$-*ribADEHC*-P$_{\text{tet}}$-RBS2-*OprF*	This study
P-RBS3	MR-1 harboring pYYDT-P$_{\text{tet}}$-*ribADEHC*-P$_{\text{tet}}$-RBS3-*OprF*	This study
P-RBS4	MR-1 harboring pYYDT-P$_{\text{tet}}$-*ribADEHC*-P$_{\text{tet}}$-RBS4-*OprF*	This study
ΔCymA	P-RBS4ΔCymA (inner-membrane cytochrome CymA deletion mutant)	Our Lab
ΔMtrC	P-RBS4ΔMtrC (outer-membrane cytochrome MtrC deletion mutant)	Our Lab
ΔOmcA	P-RBS4ΔOmcA (outer-membrane cytochrome OmcA deletion mutant)	Our Lab
ΔMtrC/ΔOmcA	P-RBS4ΔMtrC/ΔOmcA (outer-membrane cytochromes MtrC and OmcA deletion mutant)	Our Lab
**Plasmids**		
pYYDT	*pBBR1*, *kanR*, *mob*, *lacI*	Our Lab
pYYDT-P$_{\text{arcA}}$-*ribADEHC*	*pBBR1, kanR, mob, lacI, P_{arcA}-ribA-ribD-P_{arcA}-ribE-ribH-ribC*	This study
pYYDT-P$_{\text{bad}}$-*ribADEHC*	*pBBR1, kanR, mob, lacI, P_{bad}-ribA-ribD-P_{bad}-ribE-ribH-ribC*	This study
pYYDT-P$_{\text{tac}}$-*ribADEHC*	*pBBR1, kanR, mob, lacI, P_{tac}-ribA-ribD-P_{tac}-ribE-ribH-ribC*	This study
pYYDT-P$_{\text{tet}}$-*ribADEHC*	*pBBR1, kanR, mob, lacI, P_{tet}-ribA-ribD-P_{tet}-ribE-ribH-ribC*	This study
pYYDT-P$_{\text{tet}}$-*ribADEHC*- P$_{\text{tet}}$-RBS1-*OprF*	*pBBR1, kanR, mob, lacI, P_{tet}-ribA-ribD-P_{tet}-ribE-ribH-ribC-P_{tet}*-RBS1-*OprF*	This study
pYYDT-P$_{\text{tet}}$-*ribADEHC*- P$_{\text{tet}}$-RBS2-*OprF*	*pBBR1, kanR, mob, lacI, P_{tet}-ribA-ribD-P_{tet}-ribE-ribH-ribC-P_{tet}*-RBS2-*OprF*	This study
pYYDT-P$_{\text{tet}}$-*ribADEHC*- P$_{\text{tet}}$-RBS3-*OprF*	*pBBR1, kanR, mob, lacI, P_{tet}-ribA-ribD-P_{tet}-ribE-ribH-ribC-P_{tet}*-RBS3-*OprF*	This study
pYYDT-P$_{\text{tet}}$-*ribADEHC*- P$_{\text{tet}}$-RBS4-*OprF*	*pBBR1, kanR, mob, lacI, P_{tet}-ribA-ribD-P_{tet}-ribE-ribH-ribC-P_{tet}*-RBS4-*OprF*	This study

Supplementary Table 10 Results of the *S. oneidensis*-triggered RAFT polymerization mediated by the engineered *S. oneidensis* strain P-RBS4.

Entry	Time (h)	Conversion (%)	$M_{n,GPC}$ (kg mol^{-1})	$M_{n,th}$ (kg mol^{-1})	$Đ$
1	0.33	27	21.6	13.6	1.14
2	0.5	33	28.2	16.6	1.13
3	1	46	30.7	23.0	1.15
4	2	63	39.5	31.4	1.22
5	3	77	44.4	38.4	1.24
6	4	85	46.5	42.3	1.26
7	5	91	51.1	45.3	1.28

Conversion ratio was determined by ^{1}H NMR spectroscopy. Molecular weight (M_n) and polydispersity ($Đ$) were determined by GPC analysis. Polymerization conditions: After 12 hours of culturing the engineered strain P-RBS4, a certain amount of the culture fluid containing the bacteria was taken to achieve a final concentration of [OD_{600}] = 1 in the polymerization reaction mixture. [DMA]= 1 M, [CTA] = 2 mM, blue LED light (10 W, 460 nm), room temperature, 5h, cultured anaerobically with magnetic stirring. Monomer conversion determined by ^{1}H NMR. Molecular dispersity determined by GPC.

Supplementary Table 11 Examples of polymers synthesized via high-throughput *S. oneidensis*-triggered RAFT polymerization in 96 and 384-well plate.

Entry	Reaction Volume	DMA/CTA	Conversion (%)	$M_{n, th}$ (kg mol^{-1})	$M_{n, GPC}$ (kg mol^{-1})	$Đ$
1	50	200/1	99	20.2	20.5	1.21
2	200	200/1	99	20.2	21.4	1.16
3	200	100/1	99	10.2	11.4	1.13
4	200	300/1	98	29.5	33.1	1.14
5	200	500/1	97	48.6	52.3	1.21
6	200	1000/1	95	95.1	96.5	1.18
7	200	2000/1	97	194.1	199.7	1.27

Conversion ratio was determined by ^{1}H NMR spectroscopy. Molecular weight (M_n) and polydispersity ($Đ$) were determined by GPC analysis. Polymerization conditions: [riboflavin] = 10 μM, [DMA]= 1 M, blue LED light (10 W, 460 nm), room temperature, cultured aerobically with magnetic stirring. Monomer conversion determined by ^{1}H NMR. Molecular dispersity determined by GPC.

Supplementary Table 12 Primers used for RT-qPCR in this study.

Name	Sequence (5'-3')
gyrB-F	GGAACGACGGCTACCAAGA
gyrB-R	GTCAACGCACTACGGAAACC
ribA-F	TGGGTGATGTTCCATTCGGT
ribA-R	CGACCTTCAGCAGCGATTTG
ribD-F	TGGTTCTGCTGTTCACGGTT
ribD-R	GAGCGTGAGTACCACCGATT
ribE-F	TAGAACGTGCTATGGCTGCT
ribE-R	ACGAGTGATTTCAGCAGTACCA
ribH-F	TCGATGTTGCTTGGGTTCCA
ribH-R	AGTGTTAGCAGCTTGAGCGA
ribC-F	ACGGTGATAAACGTGGTCGT
ribC-R	ACCAGTTGGTGGAACGATGT
OprF-F	AAGGTGAATGGATGGCTGGT
OprF-R	ACAGCTGGACAACCGTTAGC

Supplementary References

1. Murray, A.E. *et al.* DNA/DNA hybridization to microarrays reveals gene-specific differences between closely related microbial genomes. *Proceedings of the National Academy of Sciences* **98**, 9853-9858 (2001).

2. Livak, K.J. & Schmittgen, T.D. Analysis of relative gene expression data using real-time quantitative PCR and the $2^{-\Delta\Delta CT}$ Method. *Methods* **25**, 402-408 (2001).

3. Biegasiewicz Kyle, F. *et al.* Photoexcitation of flavoenzymes enables a stereoselective radical cyclization. *Science* **364**, 1166-1169 (2019).

4. Kim, J., Nguyen, T.V.T., Kim, Y.H., Hollmann, F. & Park, C.B. Lignin as a multifunctional photocatalyst for solar-powered biocatalytic oxyfunctionalization of C–H bonds. *Nature Synthesis* **1**, 217-226 (2022).
