# Supplementary material for: Photo-excited extracellular electron transfer of electroactive microorganism triggers RAFT polymerization
Source: Nat Commun. 2025 Nov 21;16:10257. doi: 10.1038/s41467-025-65119-x (PMC12638906; doi:10.1038/s41467-025-65119-x)
Supplement: Supplementary file 2 — Description of Additional Supplementary Files [file 41467_2025_65119_MOESM2_ESM.pdf]

Description of Additional Supplementary Files

Supplementary Data 1: Computational geometries and energies of flavins.
