# Supplementary material for: Photo-excited extracellular electron transfer of electroactive microorganism triggers RAFT polymerization
Source: Nat Commun. 2025 Nov 21;16:10257. doi: 10.1038/s41467-025-65119-x (PMC12638906; doi:10.1038/s41467-025-65119-x)
Supplement: Supplementary file 4 — Reporting Summary [file 41467_2025_65119_MOESM4_ESM.pdf]

nature portfolio

Corresponding author(s): Hao Song
Last updated by author(s): Jul 22, 2025

Reporting Summary

Nature Portfolio wishes to improve the reproducibility of the work that we publish. This form provides structure for consistency and transparency in reporting. For further information on Nature Portfolio policies, see our Editorial Policies and the Editorial Policy Checklist.

Statistics

For all statistical analyses, confirm that the following items are present in the figure legend, table legend, main text, or Methods section.

n/a	Confirmed	
☐	☒	The exact sample size (*n*) for each experimental group/condition, given as a discrete number and unit of measurement
☐	☒	A statement on whether measurements were taken from distinct samples or whether the same sample was measured repeatedly
☒	☐	The statistical test(s) used AND whether they are one- or two-sided<br>*Only common tests should be described solely by name; describe more complex techniques in the Methods section.*
☒	☐	A description of all covariates tested
☒	☐	A description of any assumptions or corrections, such as tests of normality and adjustment for multiple comparisons
☐	☒	A full description of the statistical parameters including central tendency (e.g. means) or other basic estimates (e.g. regression coefficient) AND variation (e.g. standard deviation) or associated estimates of uncertainty (e.g. confidence intervals)
☒	☐	For null hypothesis testing, the test statistic (e.g. *F*, *t*, *r*) with confidence intervals, effect sizes, degrees of freedom and *P* value noted<br>*Give P values as exact values whenever suitable.*
☒	☐	For Bayesian analysis, information on the choice of priors and Markov chain Monte Carlo settings
☒	☐	For hierarchical and complex designs, identification of the appropriate level for tests and full reporting of outcomes
☒	☐	Estimates of effect sizes (e.g. Cohen's *d*, Pearson's *r*), indicating how they were calculated

Our web collection on statistics for biologists contains articles on many of the points above.

Software and code

Policy information about availability of computer code

Data collection	Nuclear magnetic resonance (1H NMR) spectroscopy of monomer/polymer solutions were measured on a Bruker AVANCE III 400 MHz. Gel permeation chromatography (GPC) was performed on a Shimadzu LC-20A system in a TSKgel guard column PWXL (6.0 mm I.D. x 4 cm) followed by a TSKgel GMPWXL (7.8 mm I.D. x 30 cm, 13 µm) or a Waters 1515 system comprises three Agilent columns (7.8 mm x 300 mm). The number-average molecular weight (Mn) of the polymers was determined by GPC of a Shimadzu LC-20A system or a Waters 1515 system. Cyclic voltametric (CV) measurements of RF and CTAs were conducted on a CHI660D electrochemical workstation (Shanghai). UV-vis absorption spectra and fluorescence spectra of RF and FMN were measured on a Thermo Scientific Varioskan LUX multimode microplate reader. The transient photoluminescence measurement of RF, FMN, and CTA1 was conducted on a time-resolved spectrometer equipped with a time correlated single photon counting (TCSPC) module (HiLight 990, Oriental Spectra). Electron spin resonance (ESR) measurement of radical polyemrization was performed using JEOL JES-FA200. Flavins (RF and FMN) were measured by using a Shimadzu LC-20A system. The geometry computations were carried out employing Gaussian 16 Rev. C.01, utilizing the Density Functional Theory (DFT) methodology for the ground state and Time-Dependent DFT (TD-DFT) for the excited state, at the B3LYP/6-31G level and the B3LYP/6-311G++(d,p) level. Real-time quantitative reverse transcription PCR (RT-qPCR) analysis of the expression level of the genes ribA, ribD, ribE, ribH, ribC, and oprF in the engineered S. oneidensis P-RBS4 and the wild-type S. oneidensis MR-1.was performed by using Power Up SYBR Green Master Mix (Thermo Fisher, A25742, USA).
Data analysis	GraphPad Prism 10.1.2, Microsoft Excel 2019, MestReNova 6.1.0-6224, Gaussian 16 Rev. C.01

For manuscripts utilizing custom algorithms or software that are central to the research but not yet described in published literature, software must be made available to editors and reviewers. We strongly encourage code deposition in a community repository (e.g. GitHub). See the Nature Portfolio guidelines for submitting code & software for further information.

Data

Policy information about availability of data

All manuscripts must include a data availability statement. This statement should provide the following information, where applicable:

- Accession codes, unique identifiers, or web links for publicly available datasets
- A description of any restrictions on data availability
- For clinical datasets or third party data, please ensure that the statement adheres to our policy

The data supporting the findings of this study are available within the paper and its Supplementary Information/Source Data files. A reporting summary for this article can also be found as a Supplementary Information file. Source data are provided with this paper.

Research involving human participants, their data, or biological material

Policy information about studies with human participants or human data. See also policy information about sex, gender (identity/presentation), and sexual orientation and race, ethnicity and racism.

Reporting on sex and gender	n/a
Reporting on race, ethnicity, or other socially relevant groupings	n/a
Population characteristics	n/a
Recruitment	n/a
Ethics oversight	n/a

Note that full information on the approval of the study protocol must also be provided in the manuscript.

Field-specific reporting

Please select the one below that is the best fit for your research. If you are not sure, read the appropriate sections before making your selection.

☒ Life sciences ☐ Behavioural & social sciences ☐ Ecological, evolutionary & environmental sciences

For a reference copy of the document with all sections, see nature.com/documents/nr-reporting-summary-flat.pdf

Life sciences study design

All studies must disclose on these points even when the disclosure is negative.

Sample size	No formal sample size calculation was performed. In line with standard practices in synthetic biology and controlled radical polymerization, we used a minimum of three biological replicates for each experiment. For each replicate, we obtained at least three independent measurements to ensure robustness and reliability of the results.
Data exclusions	No data were excluded from the analysis.
Replication	Each experiment was carried out at least three independent replications and was described in the figure legend with similar results.
Randomization	Randomization is not applicable to synthetic biology experiments, as the samples are genetically identical.
Blinding	No blinding was conducted, because blinding would not provide any reliable datasets for our all biochemical, genetic, and radical polymerization experiments. Experimental conditions were standardized to ensure consistency across all groups.

Reporting for specific materials, systems and methods

We require information from authors about some types of materials, experimental systems and methods used in many studies. Here, indicate whether each material, system or method listed is relevant to your study. If you are not sure if a list item applies to your research, read the appropriate section before selecting a response.

Materials & experimental systems

n/a	Involved in the study
☒	☐ Antibodies
☒	☐ Eukaryotic cell lines
☒	☐ Palaeontology and archaeology
☒	☐ Animals and other organisms
☒	☐ Clinical data
☒	☐ Dual use research of concern
☒	☐ Plants

Methods

n/a	Involved in the study
☒	☐ ChIP-seq
☒	☐ Flow cytometry
☒	☐ MRI-based neuroimaging

Plants

Seed stocks	Neither seed nor plants was used in our work.
Novel plant genotypes	No plants was used in our work.
Authentication	No seed was used in our work.
